# Bronchial artery embolization for the treatment of hemoptysis: permanent versus temporary embolic materials, a single center study

**DOI:** 10.1186/s42155-025-00554-x

**Published:** 2025-05-08

**Authors:** Taninokuchi Tomassoni Makoto, Perini Daniele, Porta Francesco, Braccischi Lorenzo, Zanella Sara, Basile Antonio, Modestino Francesco, Mosconi Cristina

**Affiliations:** 1 Department of Radiology, IRCCS Azienda Ospedaliero-Universitaria Di Bologna, Via Albertoni 15, Bologna, Italy; 2 Department of Medical and Surgical Sciences and Advanced Technologies, Radiodiagnostic and Radiotherapy Unit, University Hospital “Policlinico-Vittorio Emanuele”, Catania, Italy

## Abstract

**Background:**

Bronchial artery embolization (BAE) is a common interventional radiology technique used to control significant bleeding from the bronchial circulation, especially in cases of severe hemoptysis or pulmonary hemorrhage. The choice of embolizing agents plays a key role in the success, recurrence of bleeding, and safety of the procedure. However, there is no consensus on the ideal embolizing agent. This study compares the efficacy, safety, and long-term outcomes of using permanent versus temporary embolizing agents in BAE.

**Methods:**

This retrospective cohort study included patients who underwent BAE at our institution from July 2006 to May 2024. Inclusion criteria encompassed patients with hemoptysis requiring intervention, complete clinical and radiological data, and BAE with either permanent (e.g., coils, PVA particles) or temporary (e.g., gelatin sponge) embolic materials. Exclusion criteria included non-bronchial causes of hemoptysis, BAE as part of a lung transplant protocol, or use of combined embolic materials. Primary outcomes included early clinical success (cessation of bleeding during the procedure and no recurrence within a week) and late clinical success (no bleeding recurrence within 6 months). Secondary outcomes focused on procedural complications, such as pneumonia, lung infarction, or bronchial ischemia.

**Results:**

This retrospective study included a total of ninety-four procedures performed in eighty-five patients (56 males, 29 females; mean age 59; age range 8–92 years) who were admitted for BAE between July 2006 and May 2024. Permanent embolic materials were used in 59 procedures (64%), in most cases were used particles and glue (39% and 21%, respectively), while temporary embolizing materials (gelatin sponge) were used in 35 procedures (37%). Statistical analysis showed a superiority in terms of clinical outcomes in favor of permanent embolic materials (*p-*value 0,047).

**Conclusions:**

BAE is a safe procedure for control of hemoptysis of varying etiologies and possesses high rates of immediate clinical success with few complications. In terms of embolic materials, a superiority in term of late clinical success and lower hemorrhage recurrency rate with permanent materials were clearly observed in our population, with a similar safety profile. Further studies are needed to confirm our findings and strengthen evidence.

## Background

Severe hemoptysis (SH) refers to the expectoration of more than 200–600 mL of blood over 24 h or a single episode of massive bleeding that significantly impairs the patient’s ability to breathe and maintain oxygenation. SH represents a respiratory emergency with a significant mortality rate, with the literature reporting rates between 50 and 100%. Nonetheless, with timely and appropriate diagnostic and therapeutic interventions, the mortality rate can be reduced to below 20% [1].

Bronchial artery embolization (BAE) is a widely utilized interventional radiology technique primarily aimed at controlling significant bleeding from the bronchial circulation, particularly in cases of SH or hemorrhage associated with various pulmonary pathologies [2]. In the process of BAE, embolic agents are introduced into the bronchial arteries to selectively occlude them, thereby reducing blood flow to the affected region and stopping the hemorrhage [2]. The agents used for embolization play a pivotal role in determining the success of the procedure, the potential for recurrence of bleeding, and the overall safety profile. However, up to date, we have not yet reached a unanimous consensus regarding the ideal embolizing agent to be used in BAE.

By examining key clinical parameters such as embolization success rates, incidence of rebleeding, complications as well as overall patient outcomes, we hope to provide a clearer understanding of the strengths and limitations of each approach. Through this investigation, we seek to identify the optimal embolizing agent, ultimately enhancing decision-making processes and improving patient outcomes in the management of hemoptysis and other pulmonary conditions requiring BAE. This study compares the efficacy, safety, and long-term outcomes of using permanent versus temporary embolizing agents in BAE.

## Methods

### Study design

We conducted a retrospective cohort study to evaluate and compare the efficacy and safety of permanent versus temporary embolic materials used in bronchial artery embolization (BAE) for patients presenting with clinically significant hemoptysis. The study was designed and reported in accordance with the STROBE (Strengthening the Reporting of Observational Studies in Epidemiology) guidelines.

### Setting

The study was conducted at a single tertiary care university hospital (IRCCS Azienda Ospedaliero-Universitaria di Bologna, Italy), and included all eligible patients treated between July 2006 and May 2024.

### Participants

All patients who underwent BAE during the study period were screened.

Inclusion criteria were:

- Diagnosis of hemoptysis requiring interventional management
- Availability of complete clinical and radiological data
- BAE performed using either permanent (e.g., coils, polyvinyl alcohol [PVA] particles) or temporary embolic materials (e.g., gelatin sponge)

Exclusion criteria included:

- Hemoptysis due to non-bronchial causes (e.g., gastrointestinal bleeding)
- BAE performed as part of a lung transplant protocol or other major surgical procedures
- Use of both permanent and temporary embolic agents during the same procedure
- Lack of follow-up or missing data on clinical outcomes

A total of 94 procedures in 85 patients were initially reviewed; patients with missing follow-up or technical failures were excluded from the outcome analyses.

### Variables and data collection

Data were retrospectively collected from hospital electronic medical records and the radiology information system. Variables included:

- Demographic data: age, sex, smoking history, comorbidities
- Clinical presentation: hemoptysis severity (mild/moderate/massive), time from presentation to embolization
- Procedural data: embolic material used, technical success, complications
- Follow-up outcomes:
  o Early clinical success: cessation of bleeding during the procedure and no recurrence within 1 week
  o Late clinical success: absence of bleeding recurrence within 6 months
  o Complications: post-procedural pneumonia, pulmonary infarction, or bronchial ischemia

### Interventional technique

All procedures were performed by interventional radiologists with over 10 years of experience. BAE was carried out through a 5-French femoral arterial access under local anesthesia. The culprit bronchial artery was identified through bronchoscopy, contrast-enhanced CT, and angiography. Superselective catheterization was achieved using shaped catheters (e.g., cobra-type, reverse-curve) and microcatheters (1.9–2.7 F).

Embolic materials used:

- Permanent agents:
  o Microcoils (Concerto Helix, Medtronic)
  o PVA particles (Contour 500–700 µm, Boston Scientific)
  o n-Butyl Cyanoacrylate (NBCA) glue (GLUBRAN 2, GEM)
- Temporary agent:
  o Gelatin sponge (SURGISPON sheets, Capeypharma), manually cut and mixed with iodinated contrast using a dual-syringe and 3-way stopcock system to achieve the desired consistency

Technical success was defined as complete occlusion of the bleeding artery, confirmed by final angiographic images showing no distal contrast opacification.

### Bias management

To reduce measurement and classification bias, all data extraction was independently verified by two authors. We acknowledge the potential for selection bias due to the retrospective design and operator-based selection of embolic agents, which is further addressed in the Limitations section.

### Statistical analysis

Categorical variables were summarized as counts and percentages, while the numerical variable was summarized as mean and standard deviation.

Differences between the permanent and temporary embolic material groups were assessed using the Chi-square test or Fisher’s exact test for categorical variables, as appropriate. For the continuous variable, the non-parametric Wilcoxon–Mann–Whitney test was used, as the variable was not normally distributed.

A *p-*value of < 0.05 was considered statistically significant.

The analyses were carried out using IBM SPSS 27.0 (Armonk, NY: IBM Corp).

### Ethical considerations

This study was conducted in accordance with the Declaration of Helsinki. The local institutional review board waived the need for informed consent due to the retrospective design and use of anonymized data.

## Results

This retrospective study included a total of ninety-four procedures performed in eighty-five patients (56 males, 29 females; mean age 59 y; age range 8–92 years) who were admitted for BAE between July 2006 and May 2024 (Table 1).

**Table 1:** Patients’ characteristics from both groups at baseline

		Permanent embolic materials		Temporary embolic materials		
		N		N		*P-*value
Age^a^		54	57.56 ± 19.113	31	61.65 ± 17.792	0.291
Sex^b^	Male	33	61.1	23	74.2	0.245
	Female	21	38.9	8	25.8	
	Tot	54	100.0	31	100.0	
Smoke^b^	No	36	66.7	22	71.0	0.810
	Yes	18	33.3	9	29.0	
	Tot	54	100.0	31	100.0	
COPD^b^	No	50	92.6	25	80.6	0.160
	Yes	4	7.4	6	19.4	
	Tot	54	100.0	31	100.0	
Asthma^b^	No	51	94.4	31	100.0	0.297
	Yes	3	5.6	0	0.0	
	Tot	54	100.0	31	100.0	
TB^b^	No	47	87.0	30	96.8	0.248
	Yes	7	13.0	1	3.2	
	Tot	54	100.0	31	100.0	
Etiology^b^	Idiopathic	23	42.6	21	67.7	0.084
	TB	7	13.0	1	3.2	
	Pneumonia	9	16.7	1	3.2	
	COPD	4	7.4	2	6.5	
	Oncological	5	9.3	5	16.1	
	Vasc. Abnormalities	6	11.1	1	3.2	
	Tot	54	100.0	31	100.0	

^a^mean±std.dev

^b^percentages

Reasons for acute bleeding were difficult to determine due to local privacy policies, however where it has been possible to access the patient’s clinical record among the most frequent causes were infectious process (especially TB and Aspergillus), thoracic malignancies and COPD.

The right lung was affected in 56 patients (53%), the left lung in 16 patients (18%) and both lungs in 11 patients (13%). Some patients underwent rigid bronchoscopy before the intervention (*n* = 42; 44%). Contrast- enhanced computed tomography (CT) was performed to identify the feeding artery in all patients. BAE was performed under local anesthesia in all patients by five interventional radiologists with at least 10 years of experience.

### Technical and clinical success

Technical success and hemostasis were achieved in 90 procedures (95,7%), 4 procedures were unsuccessful due to anatomical reasons (tortuous bronchial artery or presence of accessory spinal artery). Embolized arteries included 58 right bronchial arteries, 13 left bronchial arteries, 5 intercostal arteries. Permanent embolic materials were used in 59 procedures (64%), in most cases were used particles and NBCA (39% and 21%, respectively), while temporary embolizing materials (gelatin sponge) were used in 35 procedures (37%).

Early clinical success data were available for 75 procedures: hemostasis was successfully achieved in 68 cases (*n* = 48 using permanent embolic materials; *n* = 20 using temporary embolic materials) but the analysis showed no statistically significant differences in terms of early clinical outcomes between the two groups (*p-*value 0,669) (Tables 2 and 3).

**Table 2:** Descriptive analyses of patients’ data grouped based on embolic material used. First-line management: Embo = embolization, FBS = fibrobronchoscopy

		Permanent embolic materials		Temporary embolic materials	
		N	Percentage (%)	N	Percentage (%)
Clinical signs of active bleeding	Mild Hemoptysis	14	26.4	13	52.0
	Moderate Hemoptysis	34	64.2	11	44.0
	Massive hemoptysis	5	9.4	1	4.0
	Tot	53	100.0	25	100.0
First-line management	Embo	26	44.1	10	28.6
	FBS	28	47.5	14	40.0
	Other	5	8.5	11	31.4
	Tot	59	100.0	35	100.0
Active bleeding at CT imaging	No	35	74.5	12	57.1
	Yes	12	25.5	9	42.9
	Tot	47	100.0	21	100.0
Active bleeding at angiography	No	22	37.9	11	31.4
	Yes	36	62.1	24	68.6
	Tot	58	100.0	35	100.0

**Table 3:** Comparison between permanent and temporary embolic materials in terms of early and late clinical success

		Permanent	Temporary	Tot	*P-value*
Early clinical success	No	4	3	7	0,699
	Yes	48	20	68	
	Tot	52	23	75	
Late clinical success	No	5	7	12	0,047
	Yes	43	16	59	
	Tot	48	23	71	

Late clinical success data were available for 71 procedures: hemostasis was successfully achieved in 59 cases (*n* = 43 using permanent embolic materials; *n* = 16 using temporary embolic materials), the analysis showed a superiority in terms of clinical outcomes in favor of permanent embolic materials (*p-*value 0,047) (Table 2).

### Complications and reintervention rate

Out of the 74 procedures whose clinical records were available, 5 were influenced by some degree of complications. Most of them were post embolization syndrome managed conservatively (*n* = 3), all of them in the permanent materials group. One patient of the permanent materials group experienced transient desaturation, resolved with oxygenotherapy the first day after the procedure. In one case, a bronchial artery rupture with periaortic hematoma was seen after contrast injection and before embolization, treated conservatively. Re-embolization on the same culprit vessel was performed in 2 cases previously treated with permanent embolic materials and in 7 cases previously treated with temporary embolic materials. One patient of each arm was retreated with surgery instead of re-embolization. A statistically significant difference between permanent and temporary materials was found regarding the reintervention rate (*p* = 0,017) (Table 4).

**Table 4:** Clinical outcomes after embolization, *p-* value refer to the comparison of the retreatment rate between temporary and permanent embolic materials

		Permanent	Temporary	Tot	*P-*value
Clinical outcomes	No rebleeding	56	27	83	0.017
	Rebleeding (Re-embolization or Surgery)	3	8	11	
	Tot	59	35	94	

## Discussion

In the field of Interventional Radiology, choosing the ideal embolizing agent in each procedure is pivotal in order to achieve an effective and safe outcome [3]. Permanent embolizing agents, are designed to create a durable and long-lasting occlusion of the targeted arteries. These materials are particularly useful when there is a need for definitive and long-term cessation of blood flow, such as in the treatment of recurrent or life-threatening bleeding episodes, or in cases of vascular malformations or tumors. Permanent agents are often preferred in situations where the embolization site is unlikely to require revascularization or follow-up interventions, and when it is critical to prevent any further blood supply to the affected area [4].

In contrast, temporary embolizing agents are designed to achieve a reversible effect on vascular occlusion. Temporary embolization is typically used in cases where acute control of bleeding is needed, but there is a possibility that the ischemic risk and subsequent necrosis of vital tissue is very high. This could occur in conditions such as bowel bleeding or other scenarios where the risk of ischemia outweighs the benefit of permanent embolization [5].

The decision to use permanent versus temporary embolizing agents in BAE is influenced by a number of factors, including the underlying pathology, the clinical presentation, the urgency of treatment, and potential risks associated with each type of agent. While permanent embolic agents are generally associated with higher success rates and lower rates of rebleeding, they come with the disadvantage of potential long-term ischemia, which can result in complications such as necrosis or impaired lung function in the treated area [6]. Nevertheless, the association between permanent agent embolization and greater complications in BAE is yet to be proven. On the other hand, temporary embolizing agents offer the advantage of reversibility, which may help avoid these long-term complications but may in theory necessitate repeated procedures if bleeding recurs or if the embolization proves incomplete [7].

Generally, four embolization materials are available for embolization of hemoptysis-associated arteries [8]. According to a recent metanalysis of Zheng et al. the most used one is gelatine sponge due to its low cost and ease of use [9]. However, according to literature, gelatin sponge tend to have a higher risk of recurrent bleeding probably due to its absorbability [10–12] thus other materials like particles and N-butyl cyanoacrylate (NBCA) glue are progressively used [9]. At our center a similar experience might be observed and nowadays we use mostly particles ang NBCA as materials of choice.

Few studies compared embolic materials in BAE in terms of efficacy [13–15] and metanalysis and literature review on materials comparison in BAE are still lacking [9]. Though in literature a number of studies have showed the efficacy and safety of permanent embolic materials used during acute hemorrhage of other districts [16].

Based on our findings we observed a statistically significant difference in terms of late clinical success between temporary and permanent embolic materials (*p-*value 0,047) in BAE. Glue and particles in our population achieved good results in terms of bleeding control and bleeding recurrency rate, showing a statistically significant difference speaking of re-embolization rate in favor of permanent embolic materials over temporary embolic materials (*p-*value 0,017) with no differences in terms of safety.

These data, aligned with the literature, show the superiority of permanent embolic materials confirming their safety and efficacy in BAE.

### Limitations

The main limitations of our study were the limited number of patients, the retrospective nature of the study and the relatively short-timed follow-up. Moreover, the choice of embolic agent was based on physician judgment, clinical scenario, and availability of catheter positioning, influencing the procedure outcomes to some extent.

## Conclusion

**Fig. 1:**
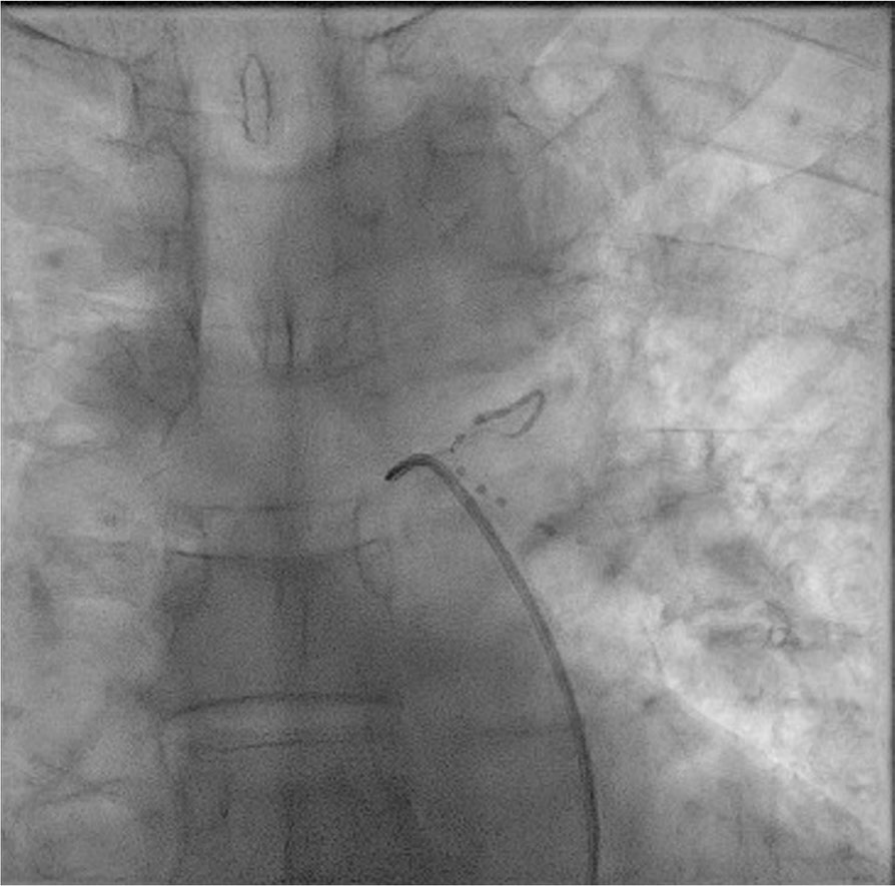
BAE using Glue as embolizing agent. Notice the radiopaque liquid agent traveling through the culprit vessel into the distal branches

BAE is a safe procedure for control of hemoptysis of varying etiologies and possesses high rates of immediate clinical success with few complications. In terms of embolic materials, a superiority in term of late clinical success and lower hemorrhage recurrency rate with permanent materials were clearly observed in our population, with a similar safety profile. Further studies are needed to confirm our findings and strengthen evidence Fig. 1.

## Data Availability

The datasets used and/or analyzed during the current study are available from the corresponding author on reasonable request.
